# Endoscopic approach to the resection of adenoid cystic carcinoma of paranasal sinuses and nasal cavity: case report and own experience

**DOI:** 10.1186/s40001-015-0189-2

**Published:** 2015-12-12

**Authors:** Piotr Wardas, Michał Tymowski, Agnieszka Piotrowska-Seweryn, Wojciech Kaspera, Aleksandra Ślaska-Kaspera, Jarosław Markowski

**Affiliations:** Clinical Department of Laryngology, School of Medicine in Katowice, Medical University of Silesia, Francuska St. 20-24, 40-029 Katowice, Poland; Department of Laryngology and ENT Oncology, Regional Hospital No. 5, Sosnowiec, Poland; Department of Neurosurgery, School of Medicine in Katowice, Medical University of Silesia, Regional Hospital No. 5, Sosnowiec, Poland

**Keywords:** Paranasal sinuses, Functional endonasal sinus surgery, Adenoid cystic carcinoma

## Abstract

Adenoid cystic carcinoma (ACC) is a rare malignant tumor that might occur in nasal cavity and paranasal sinuses. It is characteristic for poor prognosis, especially the solid histopathological subtype of the tumor. ACC might spread along nerves and fascias and it is usually diagnosed at advanced stage. Computed tomography and magnetic resonance imaging together with fine-needle biopsy are the gold standards in the diagnostic procedure of the cancer. Surgery with adjuvant therapy are the most common methods of treatment. Among the surgical approaches, the functional endonasal sinus surgery seems to be the most appropriate and favorable way of treatment. In the study, the authors present a case of a 62-year-old patient with T4aN0M0 ACC tumor treated endoscopically at the Department of Laryngology and ENT Oncology, WSS No. 5 in Sosnowiec. The authors indicate the usefulness of FESS procedure in the treatment of malignancies of nasal cavity and paranasal sinuses. They also review the recent publications on endonasal versus open approach in similar cases. In conclusions, the authors favor endonasal approach as a mini-invasive method of surgical treatment of ACC of paranasal sinuses that results in satisfactory oncological outcome and high quality of patient’s life.

## Background

Adenoid cystic carcinomas (ACC) are rare malignant tumors that usually affect salivary glands [1]. They comprise about 1 % malignancies of all head and neck cancers and about 6–10 % of malignant salivary gland tumors [2]. The most common localization for ACC is submandibular gland (15–30 %) or minor salivary glands (30 %). Involvement of nasal cavity is rare. It is much more common for maxillary sinus. ACC was first described by French scientists—Robin, Lorain and Laboulbène in 1853 and 1854 [1].

Head and neck adenoid cystic carcinoma is characteristic for spread along the nerves, high propensity for recurrence and distant metastases, which are often found when the first symptoms are observed [3–6]. It may invade the adjacent skull base by bone lysis and/or by perinervous and perivascular spread within the skull base foramina [7]. Advanced T stage reflects a poor prognosis for patients and 10-year survival is very low [1, 8]. It has been found that the histological subtype of a tumor, among which we can mention: tubular, cribriform, solid or mixed type, also influences the prognosis, giving advantage to solid subtype as the most malignant one [9, 10]. Also, some recent studies have showed that expression of c-Kit mutations is associated with a significantly poorer prognosis, while EGFR expression gives better 3-year survival [11].

The main symptoms include unilateral deformation of the nose, unilateral nasal obstruction, pain and recurrent epistaxis. Horner’s syndrome and unilateral sero-mucous otitis have been also reported [2].

Diagnosis covers ENT examination, computed tomography and obligatorily preoperative tumor biopsy. MRI scans might help in assessment of tumor extension [2].

Surgical treatment, alternatively followed by conventional or neutron irradiation, remains the optimal therapy [7]. Yet, the benefit of adjuvant radiotherapy has never been proved in randomized studies [12, 13]. Recently, the application of photodynamic therapy as adjuvant therapy to surgery in recurrent malignant tumors of the paranasal sinuses has been introduced [14]. As far as proton therapy is concerned, it should be reserved for tumors with extensions to sphenoid bone or clivus and chemotherapy for palliative treatment [2, 15].

Until, the endoscopic era lateral rhinotomy with tumor resection seemed to be the best solution. However, it is authors’ firm belief that endonasal approach can be similarly effective, resulting in complete removal with tumor-free surgical margins.

The aim of the study is to present the usefulness and radicality of FESS (functional endonasal sinus surgery) procedure as a minimally invasive one at the resection of a malignant tumor of the nasal cavity and paranasal sinuses with bone destruction.

## Case presentation

The authors report a case of a 62-year-old woman who was admitted to the Department of Neurosurgery. The patient presented with epistaxis for about 2 months, as well as nasal obstruction. Neither severe pain, nor nose deformation was reported. Anterior rhinoscopy showed the presence of tumor masses in the right nostril.

**Fig. 1:**
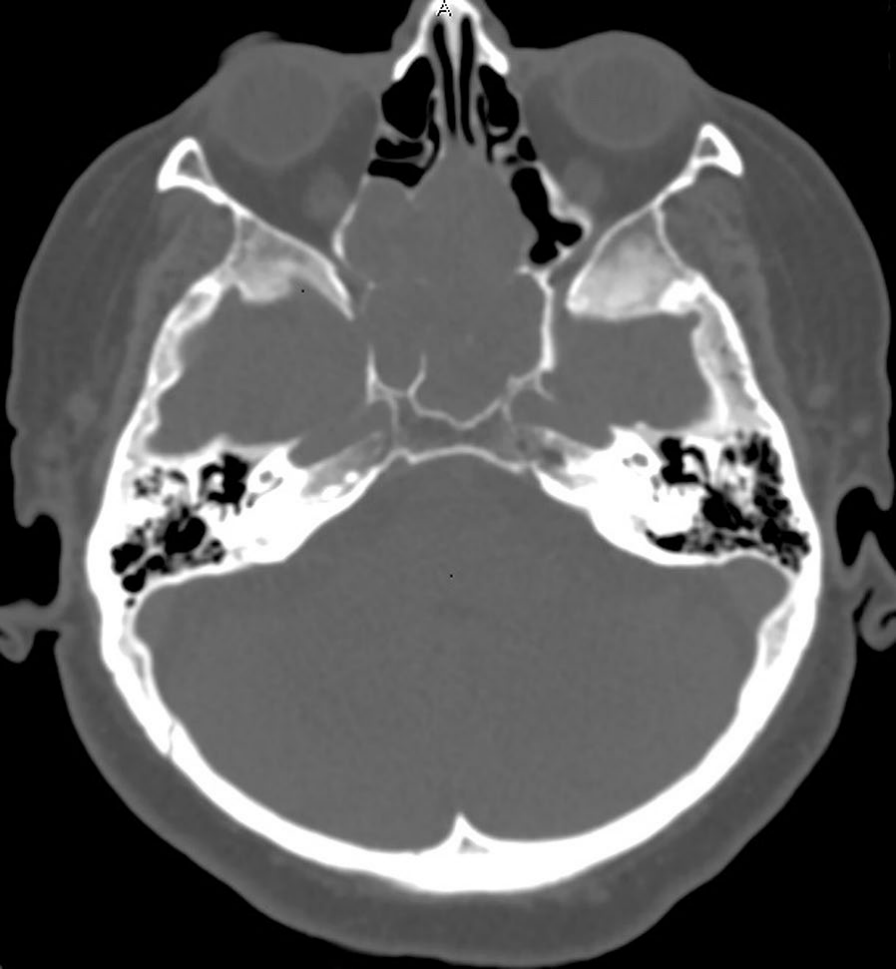
Computed tomography of paranasal sinuses. Axial projection. Hipodensive masses in the posterior ethmoid and sphenoid sinuses with expansion to nasal cavities. Partially pneumatised ethmoid cells are visible

Therefore, a computed tomography (CT) of paranasal sinuses was performed. The examination proved the presence of expansive masses in the ethmoid and sphenoid sinuses as well as nasal cavities without infiltration of the anterior cranial fossa (Fig. 1). The biopsy revealed adenoid cystic carcinoma.

**Fig. 2:**
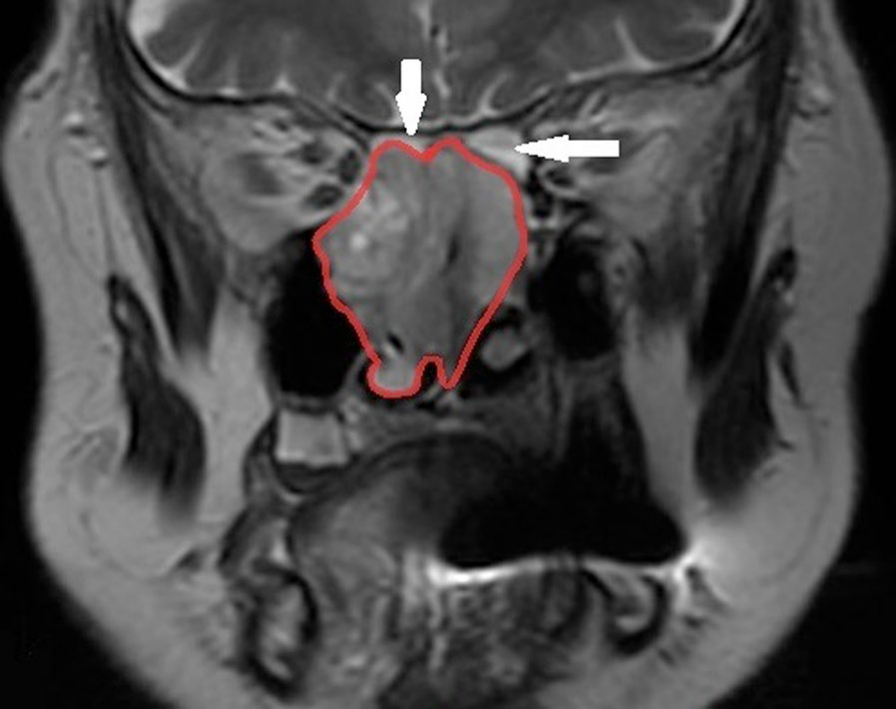
Magnetic resonance imaging. Coronal projection. Tumor masses in the right nasal cavity and bilaterally ethmoid sinuses. The *arrows* indicate ethmoids in inflammatory state, separating the tumor from anterior cranial fossa

On admission to the Department the patient was alert, logically responding, with no signs of neurological deficiencies. The patient did not suffer from blurred vision or diplopia. No allergies have been reported. Magnetic resonance imaging was performed to evaluate the expansion of the tumor (Fig. 2). The TNM staging was estimated as T4aN0M0.

Minor leukocytemia (WBC 12.08 × 10^3^/μl) and anemia (RBC 2.78 × 10^6^/μl, HGB 9.2 g/dl, HCT 27.8 %) was reported. Other laboratory tests appeared to be normal.

**Fig. 3:**
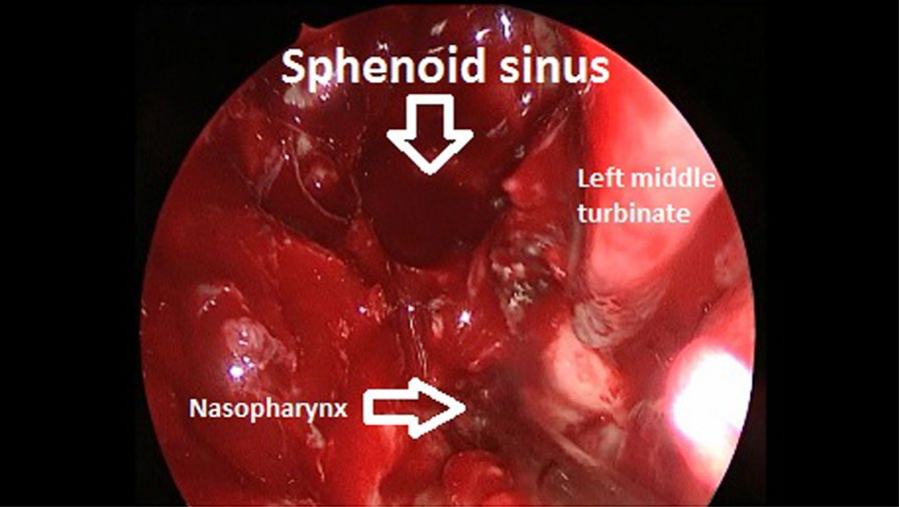
Intraoperative image after the resection of a tumor

FESS procedure in a team of neurosurgeon and ENT surgeon was performed under general anesthesia, i.e., TIVA (total intravenous anesthesia). Macroscopically a non-homogenic, cohesive, pink tumor was found. All the standard steps performed at FESS, such as uncinectomy, ethmoidectomy, sphenoidectomy, were difficult to be precisely differentiated due to massive tumor that destroyed the anatomical landmarks of this region. The operation was introduced with the resection of the central parts of the tumor masses (debulking) together with infiltrated nasal septum and frontal wall of the sphenoid sinus. This maneuver enabled identification of the probable starting point of the neoplasm, namely posterior part of nasal septum, i.e., sphenoid rostrum. Partially the margins of a tumor were visualized—tumor masses in the region of the ethmoid roof, sella turcica, clivus and left ethmoids bordered tissues free of cancerous cells. The right posterior ethmoids and lamina papyracea of the right orbit were more challenging in terms of defining exact margins of the tumor. Therefore, resection of all structures of this region, leaving bare bone of the right lamina papyracea was performed (Fig. 3). The bone itself seemed to remain unaltered. The surgeons encountered no difficulties in identification of ethmoid roof, left middle turbinate, sella turcica and other landmarks after initial debulking. No severe bleeding was noted. A dressing composed of Merocell was applicated to the nasal cavity for 48 h postoperatively. The histopathological examination revealed adenoid cystic carcinoma.

**Fig. 4:**
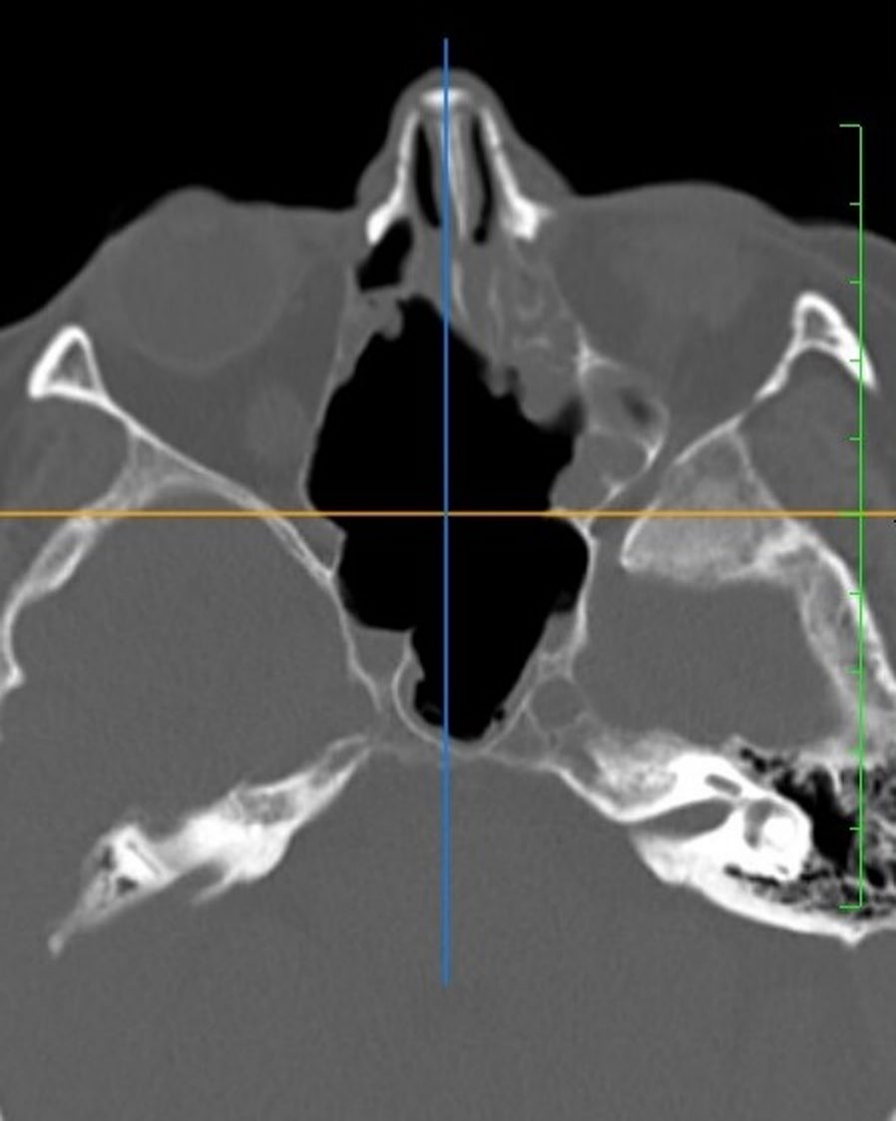
CT. Axial projection. State after radical resection of tumor masses—lack of the right superior and medial turbinate, medial wall of the right maxillary sinus, frontal walls of the sphenoid sinuses. Bare bone of the posterior part of the medial orbital wall, as mentioned in the text. The ethmoids that were well pneumatized before the surgical procedure are now filled with postoperative edematous tissues

Postoperative period was uneventful. CT scans performed after the operation showed radical resection of tumor masses (Fig. 4).

The patient was referred to an oncological department, where an adjuvant radiotherapy of total dose of 60 Gy was evaluated.

At a 2-month follow-up visit, no recurrent masses of a tumor were observed in CT (Fig. 4). The patient is regularly controlled at the outpatient clinic and in the oncological center.

## Discussion

Lack of publications on endonasal removal of an ACC of paranasal sinuses itself causes problems in thorough discussion of the mentioned topic. Most authors present general statistics on various malignant tumors treated with FESS, and few of them compare the results with open surgical approaches. Some publications refer exclusively to esthesioneuroblastoma [16–20] or melanoma of paranasal sinuses [20, 21]. The largest US database of patients (120 cases) with malignant tumors of the sinonasal tract treated with endoscopic or endoscopic-assisted resection has been reported by Hanna et al. [22].

Nevertheless, it seems that endoscopic resection of malignant tumors localized in nasal cavity and paranasal sinuses has been a matter of many controversies for the last several years [23].

Many authors believe that endoscopic sinus surgery is an effective method for sinonasal malignancies resection [23–25]. They indicate that it provides a satisfactory survival rate with few side effects and better quality of life. In addition to excellent visualization, endoscopic approaches eliminate or significantly reduce the need for craniofacial soft-tissue dissection, skeletal disassembly, and brain retraction for tumor access and resection [22]. Considering the fact that by performing FESS we avoid extensive scar that results in better esthetic effect and patient’s satisfaction, endonasal approach might be very tempting. Among open approaches Weber-Ferguson facial incision, midface degloving, craniotomy, Lynch procedure and Caldwell-Luc procedure should be mentioned [26, 27]. It is worth emphasizing that malignant character of a tumor, advanced stage of the disease leading to cachexia often deteriorates the wound healing after open surgery, sometimes resulting in nasocutaneous fistulas [14]. Such situation may extremely decrease patient’s quality of life. Also, Suh et al. claim that open surgical technique is burdened with higher risk of intra- and postoperative blood loss and higher rate of morbidity in comparison to endoscopic approach [27].

Endonasal endoscopic approach might be similarly to laryngological field beneficial in some neurosurgical operations [28–30]. For instance, Jouanneau et al. claim that the endonasal route represents an interesting alternative approach to Meckel’s cave as well as the cavernous sinus [31]. Also, according to Berhouma et al. endonasal endoscopic skull base surgery (EESBS) was initially dedicated for transsphenoid resection of pituitary adenomas [32], while nowadays it offers much more opportunities, including the treatment of clival pathologies [33]. However, some authors do not favor EESBS in the resection of malignant skull base tumors, emphasizing that such approach should be limited to certain cases [34, 35].

Most scientists state that the endonasal method is reserved for experienced surgeons and that the possibility of its application depends on TNM staging [23]. According to Hanna et al., the endoscopically treated tumors were usually of lower stage, i.e., T1–T2 [22]. They emphasize the necessity of co-operation between ENT surgeon and neurosurgeon [23]. They also focus on the role of proper adjuvant therapy, especially intensity-modulated radiation therapy (IMRT).

However, it is the matter of tumor-free margins that stimulates the greatest discussion upon endoscopic approach in the malignant tumor resection. Therefore, some authors claim that the only way to obtain above state is an open surgery with intraoperative biopsies. Yet, Sur et al. [36] suggest that the extension of the surgical resection does not affect the overall survival. The authors believe that endoscopic approach enables perfect visualization of a tumor and its borders, leading to radical resection of a tumor. In the described case tumor tissue differed from the normal mucosa to such extend that macroscopically the surgical margins seemed to be free of a tumor with high certainty.

According to Stammberger, the limitations of endoscopic technique result from the anatomical spread of the tumor, when extensive infiltration of orbit, dura/brain and other vital structures exist [23]. The key anatomical structures include internal carotid artery, optic nerve, cavernous sinuses, ethmoid roof [37]. Expansion of the tumor onto one or both cavernous sinuses is associated with the risk of injury to one of the ocular motor nerves (nerves III, IV and VI) crossing the sinus. The risk is particularly high in the case of the abducens nerve as, contrary to nerves III and IV located in the lateral wall of the cavernous sinus, this nerve passes through the sinus lumen. During endoscopic procedures in a similar way like in the open brain surgeries, ocular motor nerves can be identified by means of neurophysiological monitoring which protects them against an iatrogenic injury [38]. Nevertheless, surgical treatment of sinonasal malignancies with skull base expansion might lead to vision loss or temporary diplopia due to edema of the medial rectus muscle [14].

It is also worth mentioning that in comparison to open surgery, such as lateral rhinotomy, the dressing used after FESS is much less uncomfortable for a patient, regardless the material used (Merocell, Merogel, Nasoporin, etc.). Moreover, it is applied only for 48 h and in case of absorbable dressing, there is no need of its slightly painful removal from nostrils at all. Also, the length of hospital stay in case of endoscopic technique is shorter and the recovery is faster [37, 39].

Of course the described method does not remain without limitations and disadvantages, among which we should mention cerebrospinal fluid (CSF) leak, especially in cases with tumor extension to the skull base cavities. It seems that very little space in the described anatomical region multiplies the difficulties of dural reconstruction [39]. However, in the era of fascial or mucosal grafts in combination with tissue sealants, specialized instrumentation (curved needles, self-tying suture) an adequate reconstruction for most small defects might be obtained [39]. It has been reported that pedicled nasoseptal flaps, first introduced by Hadad in 2006 [40], reduced postoperative CSF leaks rates from 20 % to around 4 % [41, 42]. In the described case, no CSF leak was reported.

Other complications may include meningitis, epiphora in the course of dacryocystitis, mild brain contusion or pneumocephalus [22]. None of teh above was observed in our patient.

A systemic review of the recent publications on limits of endonasal endoscopic surgery in case of sinonasal tumors expanding to skull base region has been presented by Solares et al. [37]. The aspects raised in the study were: anatomical restrictions on surgical access (mentioned above), technical challenges such as dural reconstruction and hemostasis [37].

The authors are aware of the fact that the assessment of survival rate is inappropriate in such short period of time in above case, however, the aim of study was to emphasize the usefulness of FESS procedure in mini-invasive removal of paranasal sinuses’ malignancies.

## Conclusions

To sum up, the authors, who are experienced in both methods, believe that endoscopic approach in the treatment of ACC of nasal and paranasal sinuses remains an effective mini-invasive method that in well-selected patients, together with adjuvant therapies, can be satisfactorily applied, giving good results, complete removal of a tumor and high surviving rate. At the same time they emphasize the necessity of meticulous qualification to endonasal endoscopic surgeries, co-operation of ENT and neurosurgeon and the importance of application of novel technological facilities, such as neurophysiological monitoring.

## Consent

Written informed consent was obtained from the patient for publication of this Case report and any accompanying images.

## Abbreviations

ACC: adenoid cystic carcinoma
CSF: cerebrospinal fluid
CT: computed tomography
EESBS: endonasal endoscopic skull base surgery
FESS: functional endonasal sinus surgery
IMRT: intensity-modulated radiation therapy
MRI: magnetic resonance imaging
TIVA: total intravenosus anesthesia
